# Supplementary material for: Machine learning prediction of weight gain after antiretroviral therapy initiation in people with HIV: Insights from a large french real-world cohort
Source: PLoS One. 2026 Mar 6;21(3):e0344570. doi: 10.1371/journal.pone.0344570 (PMC12965677; doi:10.1371/journal.pone.0344570)
Supplement: S1 Table — INSTI, Integrase strand transfer inhibitor; TAF, Tenofovir alafenamide; NRTI, Nuclos(t)idic reverse transcriptaseinhibitor; NNRTI, Non nuclos(t)idic reverse transcriptaseinhibitor; PI, Protease inhibitor. (DOCX) [file pone.0344570.s001.docx]

**S1 Table. Predictors selection. (A) Comorbidities, (B) Co-medications and (C) Antiretroviral treatment**

| **(A)** | | | | |
| --- | --- | --- | --- | --- |
| **Comorbidities**  *Selection from the International Code of Diseases ICD-10*  [*https://icd.who.int/browse10/2019/en*](https://icd.who.int/browse10/2019/en) | | | | |
| **Predictor title** | | **ICD-10 code** | | **Code title** |
| Metabolic disorders | | E10-E14 | | Diabetes mellitus |
|  |  | E65 | | Localized adiposity |
|  |  | E66 | | Obesity |
|  |  | E74 | | Other disorders of carbohydrate metabolism |
|  |  | E78 | | Disorders of lipoprotein metabolism and other lipidaemias |
|  |  | E88 | | Other metabolic disorders |
|  |  | I10 | | Essential (primary) hypertension |
|  |  | M10 | | Gout |
| Endocrine risk factors | | E00 | | Congenital iodine-deficiency syndrome |
|  |  | E01 | | Iodine-deficiency-related thyroid disorders and allied conditions |
|  |  | E02 | | Subclinical iodine-deficiency hypothyroidism |
|  |  | E03 | | Other hypothyroidism |
|  |  | E22.0 | | Acromegaly and pituitary gigantism |
|  |  | E22.1 | | Hyperprolactinaemia |
|  |  | E24 | | Cushing syndrom |
|  |  | E28 | | Ovarian dysfunction |
|  |  | E29.1 | | Testicular hypofunction |
| Pregnancy | | Z32.1 | | Pregnancy confirmed |
|  |  | Z33 | | Pregnant state, incidental |
|  |  | Z34 | | Supervision of normal pregnancy |
|  |  | Z35 | | Supervision of high-risk pregnancy |
|  |  | O24 | | Diabetes mellitus in pregnancy |
|  |  | O26.0 | | Excessive weight gain in pregnancy |
|  |  | O30 | | Multiple gestation |
| Menopause | | N95 | | Menopausal and other perimenopausal disorders |
|  |  | E28.3 | | Primary ovarian failure |
| Sedentary lifestyle, mobility restriction | | G7 | | Diseases of myoneural junction and muscle |
|  |  | G8 | | Cerebral palsy and other paralytic syndromes |
|  |  | M15 | | Polyarthrosis |
|  |  | M16 | | Coxarthrosis |
|  |  | M17 | | Gonarthrosis |
|  |  | Z99.3 | | Dependence on wheelchair |
|  |  | Z72.3 | | Lack of physical exercise |
| Diet and hygiene habits | | F10 | | Mental and behavioural disorders due to use of alcohol |
|  |  | Z72.1 | | Alcohol use |
|  |  | K02 | | Dental caries |
|  |  | Z72.4 | | Inappropriate diet and eating habits |
|  |  | F50.4 | | Overeating associated with other psychological disturbances |
| Socio-professional risk factors | | Z72.9 | | Problem related to lifestyle, unspecified |
|  |  | Z73 | | Problems related to life-management difficulty |
|  |  | Z74 | | Problems related to care-provider dependency |
|  |  | Z75 | | Problems related to medical facilities and other health care |
|  |  | Z55 | | Problems related to education and literacy |
|  |  | Z56 | | Problems related to employment and unemployment |
|  |  | Z59 | | Problems related to housing and economic circumstances |
|  |  | Z60 | | Problems related to social environment |
|  |  | Z65 | | Problems related to other psychosocial circumstances |
| Malnutrition | | E40 | | Kwashiorkor |
|  |  | E41 | | Nutritional marasmus |
|  |  | E42 | | Marasmic kwashiorkor |
|  |  | E43 | | Unspecified severe protein-energy malnutrition |
|  |  | E44 | | Protein-energy malnutrition of moderate and mild degree |
|  |  | E46 | | Unspecified protein-energy malnutrition |
| Thyrotoxicosis | | E05 | | Thyrotoxicosis |
| Anorexia | | F50.0 | | Anorexia nervosa |
|  |  | F50.1 | | Atypical anorexia nervosa |
| Intake of toxic substances | | F11 | | Mental and behavioural disorders due to use of opioids |
|  |  | F14 | | Mental and behavioural disorders due to use of cocaine |
|  |  | F15 | | Mental and behavioural disorders due to use of other stimulants, including caffeine |
| Mood disorders | | F06.3 | | Organic mood [affective] disorders |
|  |  | F06.4 | | Organic anxiety disorder |
|  |  | F06.6 | | Organic emotionally labile [asthenic] disorder |
|  |  | F3 | | Mood [affective] disorders |
|  |  | F40 | | Phobic anxiety disorders |
|  |  | F41 | | Other anxiety disorders |
|  |  | F43 | | Reaction to severe stress, and adjustment disorders |
|  |  | F48 | | Other neurotic disorders |
|  |  | Z91.5 | | Personal history of self-harm |
|  |  | Z91.4 | | Personal history of psychological trauma, not elsewhere classified |
|  |  | X84 | | Intentional self-harm by unspecified means |
|  |  | X7 | | Intentional self-harm |
| Schizophrenia disorders | | F2 | | Schizophrenia, schizotypal and delusional disorders |
| Bulimia | | F50.2 | | Bulimia nervosa |
|  |  | F50.3 | | Atypical bulimia nervosa |
| Non-compliance with treatment | | Z91.1 | | Personal history of noncompliance with medical treatment and regimen |
| Sleep disorders | | F51 | | Nonorganic sleep disorders |
|  |  | G47 | | Sleep disorders |
| Enterocolitis | | K50 | | Crohn disease |
|  |  | K51 | | Ulcerative colitis |
|  |  | K52 | | Other noninfective gastroenteritis and colitis |
|  |  | K55 | | Vascular disorders of intestine |
|  |  | K58 | | Irritable bowel syndrome |
|  |  | K59 | | Other functional intestinal disorders |
|  |  | K63.8 | | Other specified diseases of intestine |
|  |  | K63.9 | | Disease of intestine, unspecified |
|  |  | K90 | | Intestinal malabsorption |
| Stomies | | Z93.1 | | Gastrostomy status |
|  |  | Z93.2 | | Ileostomy status |
|  |  | Z93.3 | | Colostomy status |
|  |  | Z93.4 | | Other artificial openings of gastrointestinal tract status |
| **(B)** | | | | |
| **Co-medications**  *Selection from international non-proprietary names (INNs) and specialty names*  *[https://base-donnees-publique.medicaments.gouv.fr/index.php#result](https://base-donnees-publique.medicaments.gouv.fr/index.php#result)* | | | | |
| **Predictor title** | | **INN** | | **Specialty names containing the molecule** |
| Atypical neuroleptics | | Risperidone | | Risperdal |
|  |  | Aripiprazole | | Abilify |
|  |  | Amisulpride | | Solian |
|  |  | Clozapine | | Léponex |
|  |  | Quetiapine | | Xeroquel |
|  |  | Olanzapine | | Zyprexa, Zalasta, Zypadhera |
|  |  | Pimozide | | Orap |
|  |  | Paliperidone | | Xeplion, Trevicta |
| Tricyclic antidepressants | | Imipramine | | Tofranil |
|  |  | Amitriptyline | | Laroxyl, Elavil |
|  |  | Clomipramine | | Anafranil |
|  |  | Doxépine | | Quitaxon |
|  |  | Amoxépine | | Défanyl |
| Other antidepressants | | Paroxétine | | Déroxat, Divarius |
|  |  | Miansérine | | Athymil |
|  |  | Mirtazapine | | Norset |
| Thymoregulators and anti-convulsants | | Lithium | | Téralithe |
|  |  | Valproate | | Dépakine Dépakote, Micropakine |
|  |  | Gabapentine | | Neurontin |
|  |  | Carbamazépine | | Tégrétol |
| Corticosteroids | | Prednisone | | Cortancyl |
|  |  | Prednisolone | | Solupred |
|  |  | Méthylprednisolone | | Médrol |
| GLP1 analogues | | Dulaglutide | | Trulicity |
|  |  | Liraglutide | | Victoza, Saxenda, Xultophy |
|  |  | Sémaglutide | | Ozempic |
|  |  | Exénatide | | Byetta, Byduréon |
| Medications associated with weight loss (others) | | Benfluorex | | Médiator |
|  |  | Naltrexone | | Révial, Rélistor |
|  |  | Bupropion | | Zyban |
|  |  | Topiramate | | Epitomax |
|  |  | Orlistat | | Xénical |
| **(C)** | | | | |
| **Antiretroviral treatment**  *Selection from international non-proprietary names (INNs) and specialty names*  *[https://base-donnees-publique.medicaments.gouv.fr/index.php#result](https://base-donnees-publique.medicaments.gouv.fr/index.php#result)* | | | | |
| **Predictor title** | **INN** | | **Specialty names containing the molecule** | |
| Second-generation INSTI | Dolutegravir | | Tivicay, Juluca, Dovato, Triumeq, Gsk2619619 | |
|  | Bictegravir | | Biktarvy | |
|  | Cabotegravir | | Vocabria, Cabenuva, Gsk1265744 | |
| INSTI | Raltegravir | | Isentress | |
|  | Elvitegravir | | Genvoya, Stribild | |
|  | Dolutegravir | | Tivicay, Juluca, Dovato, Triumeq, Gsk2619619 | |
|  | Bictegravir | | Biktarvy | |
|  | Cabotegravir | | Vocabria, Cabenuva, Gsk1265744 | |
| TAF | Tenofovir alafénamide | | Odefsey, Genvoya, Descovy, Biktarvy, Vemlidy, Symtuza | |
| NRTI | Zidovudine | | Rétrovir, Combivir, Trizivir | |
|  | Lamivudine | | Epivir, Zeffix, Combivir, Delstrigo, Dovato, Kivexa, Triumeq, Trizivir, Gsk2619619 | |
|  | Abacavir | | Ziagen, Kivexa, Triumeq, Trizivir, Gsk2619619 | |
|  | Tenofovir disoproxil fumarate | | Viread, Atripla, Delstrigo, Evipléra, Stribild, Truvada, Complera | |
|  | Emtricitabine | | Emtriva, Atripla, Biktarvy, Descovy, Eviplera, Genvoya, Odefsey, Stribild, Truvada, Symtuza, Complera | |
|  | Tenofovir alafénamide | | Odefsey, Genvoya, Descovy, Biktarvy, Vemlidy, Symtuza | |
|  | Stavudine | | Zerit | |
|  | Didanosine | | Videx | |
| NNRTI | Nevirapine | | Viramune | |
|  | Efavirenz | | Atripla, Sustiva | |
|  | Etravirine | | Intelence | |
|  | Rilpivirine | | Edurant, Eviplera, Juluca, Odefsey, Rekambys, Complera, Cabenuva, tmc278 | |
|  | Doravirine | | Pifeltro, Desltrigo, Mk1439 | |
| PI | Saquinavir | | Invirase | |
|  | Ritonavir | | Norvir, Kaltra | |
|  | Lopinavir | | Kaletra | |
|  | Atazanavir | | Reyataz | |
|  | Fosamprenavir | | Telzir | |
|  | Tipranavir | | Aptivus | |
|  | Darunavir | | Prézista, Prezcobix, Rézolsta | |
|  | Indinavir | | Crixivan | |
| Maraviroc | Maraviroc | | Celsentri | |
| Islatravir | Islatravir | | Mk8591 | |
| Bms955176 | Bms955176 | | Bms955176 | |

INSTI, Integrase strand transfer inhibitor; TAF, Tenofovir alafenamide; NRTI, Nuclos(t)idic reverse transcriptaseinhibitor; NNRTI, Non nuclos(t)idic reverse transcriptaseinhibitor; PI, Protease inhibitor.
