# Supplementary material for: Machine learning prediction of weight gain after antiretroviral therapy initiation in people with HIV: Insights from a large french real-world cohort
Source: PLoS One. 2026 Mar 6;21(3):e0344570. doi: 10.1371/journal.pone.0344570 (PMC12965677; doi:10.1371/journal.pone.0344570)
Supplement: S2 Table — (DOCX) [file pone.0344570.s002.docx]

**S2 Table. Hyperparameters setting for final XGBoost models. The following hyperparameters were tuned using grid search with 10-fold cross-validation via tidymodels package.**

| Hyperparameter (R name) | XGBoost equivalent | Definition | Final Value M6 | Final Value M12 | Final Value M24 |
| --- | --- | --- | --- | --- | --- |
| trees | n_estimators | Number of trees in the ensemble | 500 | 500 | 500 |
| tree_depth | max_depth | Maximum depth of a tree | 3 | 3 | 2 |
| learn_rate | eta | Step size shrinkage used in update | 0,0114636 | 0,0139337 | 0,0602089 |
| min_n | min_child_weight | Minimum number of data points in a node | 19 | 30 | 3 |
| stop_iter | early_stopping_rounds | Criteria of early stopping | 6 | 18 | 14 |
| sample_size | subsample | Subsample ratio of the training instance | 0,9576271 | 0,9237288 | 0.6271186 |
| mtry | colsample_bytree | Number of predictors sampled at each split | 110 | 81 | 74 |
