# Supplementary material for: Machine learning prediction of weight gain after antiretroviral therapy initiation in people with HIV: Insights from a large french real-world cohort
Source: PLoS One. 2026 Mar 6;21(3):e0344570. doi: 10.1371/journal.pone.0344570 (PMC12965677; doi:10.1371/journal.pone.0344570)
Supplement: S3 Table — (DOCX) [file pone.0344570.s003.docx]

**S3 Table.** **Tables from Dat’AIDS database.**

| **Table**  **(Rows x columns)** | **Patients (n)** | **Missing data (%)** | **Comments** |
| --- | --- | --- | --- |
| **DATA**  (78,621x38) | 78 621 | 15,32 | Reference table for socio-demographic characteristics of the cohort |
| **ANTECEDENTS**  (1,944,985x5) | 78 327 | 10,69 | 39,627 patients had a history of interest for the study (see predictors linked to comorbidities) |
| **COMEDIC**  (6,297,982x4) | 69 458 | 0,00 | 9,954 patients presented a comedication of interest for the study (see predictors linked to comedications) |
| **CVVIH**  (2,704,230x6) | 77 858 | 3,17 | 77,413 patients remaining after data cleaning and removal of outliers |
| **CD4CD8**  (2,840,548x7) | 77 902 | 10,70 | 76 743 patients remaining after data cleaning and removal of outliers |
| **LEUCO**  (2,228,821x7) | 74 879 | 1,79 | Not retained for the study |
| **CREAT**  (2,059,822x18) | 74 692 | 13.04 | Not retained for the study |
| **TRANSA**  (4,371,096x8) | 75 985 | 0,03 | Not retained for the study |
| **LIPIDES**  (3,912,458x8) | 73 338 | 0,04 | Not retained for the study |
| **EXAM_CLINIQUE**  (3,106,170x10) | 78 665 | 43,93 | There are more patients in this table than in the “DATA” base table  60,223 patients had at least one weight recorded in the follow-up |
| **EVT_THER**  (462,611x5) | 78 665 | 3,01 | There are more patients in this table than in the “DATA” base table |
