# Supplementary material for: Machine learning prediction of weight gain after antiretroviral therapy initiation in people with HIV: Insights from a large french real-world cohort
Source: PLoS One. 2026 Mar 6;21(3):e0344570. doi: 10.1371/journal.pone.0344570 (PMC12965677; doi:10.1371/journal.pone.0344570)
Supplement: S4 Table — Values in number (%), 1Average (quartiles). †Not provided: 537 (8.04), ‡Not provided: 410 (7.23), §Not provided: 285 (6.03). $Not specified: 530 (8.10), $$Not specified: 409 (7.31), $$$Not specified: 284 (6.06). (DOCX) [file pone.0344570.s004.docx]

**S4 Table. Characteristics of the subpopulations of PLHIV treated in the first line used for weight prediction at 6, 12 and 24 months: maximum variation in weight per month limited to 5 kg (A) and 3 kg (B).**

| **(A)**  *Limited to 5 kg / month* | **Cohort M6**  **N = 6679** | **Cohort M12**  **N = 5670** | **Cohort M24**  **N = 4726** |
| --- | --- | --- | --- |
| **Age (year)^1^** | 40.26 (31.20-48.10) | 41.01 (32.10-49.00) | 42.06 (33.20-50.10) |
| **Gender** | Male: 4815 (72.09)  Female: 1819 (27.23)  Trans M-F: 45 (0.67) | Male: 4158 (73.33)  Female: 1473 (25.98)  Trans M-F: 39 (0.69) | Male: 3478 (73.59)  Female: 1215 (25.71)  Trans M-F: 33 (0.70) |
| **Birth country** | † | ‡ | § |
| *France* | 3729 (55.83) | 3261 (57.51) | 2719 (57.53) |
| *West and Central Africa* | 1387 (20.76) | 1138 (20.07) | 969 (20.50) |
| *Middle East and North Africa* | 242 (3.62) | 198 (3.49) | 183 (3.87) |
| *Others* | 784 (11.74) | 663 (11.69) | 570 (12.06) |
| **VIH-1** | 6638 (99.39) | 5645 (99.56) | 4697 (99.37) |
| **AIDS stage** | 880 (13.18) | 722 (12.73) | 537 (11.36) |
| **Weight at first visit (kg)^1^** | 70.78 (62.00-78.00) | 71.08 (62.00-78.50) | 71.52 (63.00-79.00) |
| **Weight at checkpoint (kg)^1^** | 72.23 (63.00-80.00) | 73.11 (64.00-81.00) | 73.98 (64.00-82.00) |
| **Viral load (log)^1^** | 1.68 (1.30-1.67) | 1.67 (1.30-1.60) | 1.66 (1.30-1.60) |
| **CD4 count (/mm3) ^1^** | 573 (379-711) | 606 (405-748) | 647 (447-797) |
| **CD4CD8 ratio^1^** | 0.80 (0.46-1.02) | 0.86 (0.51-1.11) | 0.95 (0.58-1.21) |
| **ARV treatment** |  |  |  |
| *TAF* | 590 (8.83) | 542 (9.56) | 461 (9.75) |
| *INSTI* | 1684 (25.21)  *Dont INI de deuxième génération : 872 (13.06)* | 1410 (24.87)  *Dont INI de deuxième génération : 746 (13.16)* | 1197 (25.33)  *Dont INI de deuxième génération : 678 (14.35)* |
| *NRTI* | 6514 (97.53) | 5545 (97.80) | 4620 (97.76) |
| *NNRTI* | 1463 (21.90) | 1387 (24.46) | 1350 (28.57) |
| *PI* | 3522 (52.73) | 2838 (50.05) | 2162 (45.75) |
| **≥ 1 Supposed comorbidities of weight gain** | 1240 (18.57) | 1149 (20.26) | 1063 (22.49) |
| **≥ 1** **Supposed comorbidities of weight loss** | 121 (1.81) | 104 (1.83) | 105 (2.22) |
| **≥ 1 Supposed comorbidities of weight variation** | 772 (11.56) | 759 (13.39) | 662 (14.01) |
| **≥ 1 Supposed comedications of weight gain** | 202 (3.02) | 168 (2.96) | 173 (3.66) |
| **≥ 1 Supposed comedications of weight loss** | 4 (0.06) | 2 (0.04) | 9 (0.19) |

| **(B)**  *Limited to 3 kg / month* | **Cohort M6**  **N = 6540** | **Cohort M12**  **N = 5596** | **Cohort M24**  **N = 4684** |
| --- | --- | --- | --- |
| **Age (year)^1^** | 40.28 (31.20-48.20) | 40.97 (32.10-49.00) | 42.06 (33.20-50.10) |
| **Gender** | Male : 4724 (72.23)  Female : 1773 (27.11)  Trans M-F : 43 (0.66) | Male : 4107 (73.39)  Female : 1450 (25.91)  Trans M-F : 39 (0.70) | Male : 3443 (73.51)  Female : 1208 (25.79)  Trans M-F : 33 (0.70) |
| **Birth country** | $ | $$ | $$$ |
| *France* | 3660 (55.97) | 3219 (57.52) | 2693 (57.49) |
| *West and Central Africa* | 1336 (20.43) | 1118 (19.98) | 961 (20.52) |
| *Middle East and North Africa* | 236 (3.61) | 195 (3.48) | 182 (3.89) |
| *Others* | 778 (11.90) | 655 (11.79) | 564 (12.04) |
| **VIH-1** | 6504 (99.45) | 5571 (99.55) | 4655 (99.38) |
| **AIDS stage** | 848 (12.97) | 706 (12.62) | 530 (11.32) |
| **Weight at first visit (kg)^1^** | 70.86 (62.00-78.00) | 71.67 (62.00-79.00) | 71.64 (63.00-79.58) |
| **Weight at checkpoint (kg)^1^** | 72.12 (63.00-80.00) | 73.07 (64.00-81.00) | 73.98 (64.00-82.00) |
| **Viral load (log)^1^** | 1.68 (1.30-1.67) | 1.67 (1.30-1.60) | 1.65 (1.30-1.60) |
| **CD4 count (/mm3) ^1^** | 574 (379-711) | 608 (406-750) | 646 (446-797) |
| **CD4CD8 ratio^1^** | 0.79 (0.46-1.02) | 0.87 (0.51-1.12) | 0.95 (0.58-1.21) |
| **ARV treatment** |  |  |  |
| *TAF* | 576 (8.81) | 534 (9.54) | 454 (9.70) |
| *INSTI* | 1652 (25.26)  *Dont INI de deuxième génération : 853 (13.04)* | 1396 (24.95)  *Dont INI de deuxième génération : 738 (13.19)* | 1183 (25.26)  *Dont INI de deuxième génération : 671 (14.33)* |
| *NRTI* | 6380 (97.53) | 5475 (97.84) | 4578 (97.74) |
| *NNRTI* | 1443 (22.06) | 1367 (24.43) | 1343 (28.67) |
| *PI* | 3437 (52.55) | 2798 (50.00) | 2143 (45.75) |
| **≥ 1 Supposed comorbidities of weight gain** | 1209 (18.49) | 1129 (20.18) | 1053 (22.48) |
| **≥ 1** **Supposed comorbidities of weight loss** | 118 (1.80) | 101 (1.80) | 103 (2.20) |
| **≥ 1 Supposed comorbidities of weight variation** | 770 (11.77) | 749 (13.38) | 657 (14.03) |
| **≥ 1 Supposed comedications of weight gain** | 191 (2.92) | 164 (2.93) | 171 (3.65) |
| **≥ 1 Supposed comedications of weight loss** | 4 (0.06) | 1 (0.02) | 6 (0.13) |

Values in number (%), ^1^Average (quartiles)

†Not provided: 537 (8.04), ‡Not provided: 410 (7.23), §Not provided: 285 (6.03)

$Not specified: 530 (8.10), $$Not specified: 409 (7.31), $$$Not specified: 284 (6.06).
