# Supplementary material for: Machine learning prediction of weight gain after antiretroviral therapy initiation in people with HIV: Insights from a large french real-world cohort
Source: PLoS One. 2026 Mar 6;21(3):e0344570. doi: 10.1371/journal.pone.0344570 (PMC12965677; doi:10.1371/journal.pone.0344570)
Supplement: S6 Table — aValue obtained after 10 cross-validation. The RMSE evaluates the accuracy of the model by measuring the average difference between the actual values and the predictions (the lower it is, the better the performance of the model). The R2 of RMSE measures the quality of fit of the model in relation to the variability of the real data (0: no fit, 1: perfect fit). The relative RMSE expresses the relative error of the mean compared to the actual values. Relative bias measures the systematic error of the model compared to actual values. (DOCX) [file pone.0344570.s006.docx]

**S6 Table. Performance of XGBoost models for weight prediction at 6, 12 and 24 months in study subpopulations. (A) Subpopulation limited to 5kg/month, (B) Subpopulation limited to 3 kg/month.**

| (A) | *Subpopulation limited to 5 kg / month* | | |
| --- | --- | --- | --- |
|  | *Prediction M6*  *^N= 6679^* | *Prediction M12*  *^N= 5670^* | *Prediction M24*  *^N= 4726^* |
| RMSE, kg^a^ | 4.42 | 4.98 | 5.96 |
| R2 of RMSE^a^ | 0.896 | 0.877 | 0.824 |
| Relative RMSE, % | 6.21 | 6.81 | 7.70 |
| Relative biais, % | 0.13 | 0.16 | -0.02 |
|  |  |  |  |
| (B) | ***Subpopulation limited to 3 kg / month*** | | |
|  | *Prediction M6*  *^N= 6540^* | *Prediction M12*  *^N= 5596^* | *Prediction M24*  *^N= 4684^* |
| RMSE, kg^a^ | 3.89 | 4.84 | 5.93 |
| R2 of RMSE^a^ | 0.920 | 0.881 | 0.842 |
| Relative RMSE, % | 5.35 | 6.49 | 7.65 |
| Relative biais, % | -0.10 | 0.40 | 0.29 |

^a^ Value obtained after 10 cross-validation.

The RMSE evaluates the accuracy of the model by measuring the average difference between the actual values and the predictions (the lower it is, the better the performance of the model). The R2 of RMSE measures the quality of fit of the model in relation to the variability of the real data (0: no fit, 1: perfect fit). The relative RMSE expresses the relative error of the mean compared to the actual values. Relative bias measures the systematic error of the model compared to actual values.
