# Supplementary material for: Machine learning prediction of weight gain after antiretroviral therapy initiation in people with HIV: Insights from a large french real-world cohort
Source: PLoS One. 2026 Mar 6;21(3):e0344570. doi: 10.1371/journal.pone.0344570 (PMC12965677; doi:10.1371/journal.pone.0344570)
Supplement: Attachment — Submitted filename: PlosOne.pdf [file pone.0344570.s007.pdf]

This study uses machine learning (XGBoost) on a large French HIV cohort to predict weight gain after antiretroviral therapy (ART) initiation at 6, 12, and 24 months using 112 baseline clinical variables. The models marginally outperformed a simple benchmark (baseline weight) but did not achieve clinically actionable accuracy, primarily due to data heterogeneity and absence of behavioral variables.

Major Points

- Innovation and Importance:
  The study addresses a well-recognized clinical issue—excessive weight gain following ART—and applies advanced ML methods on a large real-world dataset, filling a gap in prediction research in HIV care.
- Sample and Data:
  The cohort size is impressive (over 24,000 ART-naïve adults), and the data includes a comprehensive range of clinical, laboratory, and demographic features.
- Model Choice:
  XGBoost is an appropriate algorithm given the dataset size and the mix of variable types. The use of cross-validation and careful train/test splitting is appropriate.
- Limitations Clearly Stated:
  The paper explicitly acknowledges limitations around the lack of high-quality behavioral, lifestyle, and granular longitudinal data. It also carefully details the reduction of sample size due to missing data and the impact such missingness has on model performance.
- Performance and Interpretation:
  The models achieve RMSE values of 4.6, 5.3, and 6.4 kg (at 6, 12, and 24 months), which is only a marginal improvement over the baseline. Baseline weight overwhelms all other predictors; other variables, including ART components, have limited additional value. The discussion around why ML does not perform well in this context is appropriately critical and balanced.
- Methodological Transparency:
  Data processing, variable selection, and imputation steps are described transparently. Sensitivity analyses excluding outliers and restricting datasets were thoughtful and further contextualized the findings.
- Ethical and Data Sharing Declarations:
  Ethical approvals and data access limitations are well described. The paper observes regulatory limitations on data sharing, and this is explained up front.

Minor Points

- Lifestyle Predictor Handling:
  Lifestyle and behavioral factors are mentioned as potentially important, but their absence is only briefly discussed. The authors could speculate more on how to either estimate or collect these for future work.
- Imputation Strategy:
  The choice of k-nearest neighbors for imputation is standard, but potential biases from this method are not deeply explored. Some simulation or secondary analysis around missingness mechanisms might add value.

- Model Calibration:
  The paper does not report calibration plots (e.g., observed vs predicted weights). Given the clinical implications, calibration is important to assess and could be shown, even if limited.
- External Generalizability:
  The study's scope is the French population, but some comment about applicability to other settings, especially outside Europe, would be welcome.
- Figure/Table Presentation:
  Figures and tables are referenced well, but future submissions could improve access to the key visuals (since they are in supplemental content).
- Comparison to Published Literature:
  Only a few related studies are referenced (notably Motta et al.). Adding further international context may help underscore the universality of the limitations found.

Overall Assessment

This is a high-quality, carefully executed study with an honest appraisal of the challenges of applying ML to real-world clinical prediction in HIV. While negative in primary results, the findings are valuable and relevant to the field. The main area for improvement would be a deeper exploration of missing data and model calibration, and a more detailed discussion of the challenges of integrating behavioral variables in future iterations.
